# Incorporating Carbamate Functionalities in Multifunctional Monomer System Enhances Mechanical Properties of Methacrylate Dental Adhesives

**DOI:** 10.3390/polym17202780

**Published:** 2025-10-17

**Authors:** Burak Korkmaz, Erhan Demirel, Anil Misra, Candan Tamerler, Paulette Spencer

**Affiliations:** 1 Institute for Bioengineering Research, University of Kansas, 1530 W. 15th Street, Lawrence, KS 66045-7608, USA; korkmazb@ku.eductamerler@ku.edu (C.T.); 2 Department of Chemistry, Faculty of Science and Letters, Istanbul Technical University, Maslak, 34469 Istanbul, Turkey; 3 Department of Civil and Environmental Engineering, Florida International University, 10555 W. Flagler Street, Miami, FL 33174-1630, USA; anmisra@fiu.edu; 4 Department of Mechanical Engineering, University of Kansas, 1530 W. 15th Street, Lawrence, KS 66045-7608, USA; 5 Bioengineering Program, University of Kansas, 1530 W. 15th Street, Lawrence, KS 66045-7608, USA

**Keywords:** dental materials, long chain monomer, hydrogen bonding, carbamate, sol–gel reaction, dynamic mechanical analysis

## Abstract

Although resin-based composite is the most popular direct restoration material in the U.S., composite restorations can fail shortly after placement. The leading cause of failure is recurrent marginal decay. The adhesive that bonds the composite to the tooth is intended to seal the margin, but the degradation of the adhesive seal to dentin leads to gaps that are infiltrated by cariogenic bacteria. The development of strategies to mitigate adhesive degradation is an area of intense interest. Recent studies focus on exploiting hydrogen–bond interactions to enhance polymer network stability. This paper presents the preparation and characterization of model adhesives that capitalize on carbamate-functionalized long-chain silane monomers to enhance polymer stability and mechanical properties in wet environments. The adhesive composition is HEMA/BisGMA, 3-component photoinitiator system, carbamate-functionalized long-chain silane monomers, e.g., commercial SHEtMA (Cb1) and newly synthesized SHEMA (Cb2). Polymerization behavior, water sorption, leachates, and dynamic mechanical properties were investigated. The properties of Cb1 and Cb2 were compared to previously studied middle- (SC4) and short-chain (SC5) silane monomers. Cb1- and Cb2-formulations exhibit greater resilience under wet conditions as compared to middle-chain silane monomers. Dental adhesives containing the carbamate-functionalized long-chain silane monomers exhibit reduced flexibility in water-submersed conditions and enhanced stability as a result of increased hydrogen–bond interactions. The results emphasize the critical role of hydrogen bonding in maintaining structural integrity of dental adhesive formulations under conditions that simulate the wet, oral environment.

## 1. Introduction

Resin-based composites are the most popular and commonly used direct restoration material in the United States [1]. The popularity is traced to their esthetic appeal, conserving tooth structure, and environmental benefits [2]. In spite of the popularity and benefits, composite restorations can fail shortly after placement [3]. A recent clinical study reported that out of 700,000 posterior composite restorations placed in U.S. general dentistry practices, more than 20,000 failed in the first year and over 10% failed within 5 years [1]. Nearly 70% of composite restorations are replacements for failed resin restorations [4]. The cycle of repeated composite restoration replacement is a pernicious problem. Each replacement risks pulpal injury, weakens the tooth, and eventually leads to tooth loss [4].

The leading cause of composite restoration failure is secondary caries, i.e., recurrent decay at the composite/tooth margin [1,4,5,6,7,8,9]. Composite materials lack the inherent capacity to seal gaps, voids, and discrepancies at the composite/tooth margin. The adhesive that bonds the composite to the tooth surface is intended to seal the margin, but the adhesive seal to dentin is readily degraded by enzymes, acids, oral fluids, masticatory stress, and combinations of these factors [2,3,6,10,11,12,13,14,15,16,17,18,19,20,21]. Cariogenic bacteria such as *Streptococcus mutans* (*S. mutans*) infiltrate the breached interfacial seal [3,4,6,8,22]. The destruction provoked by the infiltrating bacteria and the subsequent release of bacterial by-products leads to further erosion of the adhesive, demineralization and decomposition of the tooth, and enlarged marginal gaps [4,9]. The degradation of the composite/tooth margin where the adhesive is applied leads to secondary caries, fracture, detachment, and failure of the composite restoration [4,17].

There have been significant advances in adhesive dentistry over the past several decades, and these advances have enhanced our understanding of how the harsh oral environment degrades most dental resins. The advances have also fueled diverse strategies to mitigate adhesive degradation. The strategies include approaches to protect and/or reinforce the adhesive/dentin interface with novel monomers [15,19], Zn-containing polydopamine-based materials [21] and tethered functionalized peptides to inhibit bacteria and/or repair damaged dentin [23,24]. The strategies include the development and investigation of diverse monomers to increase hydrolytic resistance, reduce phase separation, provide antibacterial properties, and increase durability [2,6,15,17,19,25,26].

Dental adhesives are generally composed of a blend of hydrophobic and hydrophilic monomers, photoinitiators, solvents, and other additives [12]. The hydrophobic monomers are predominantly dimethacrylates [27]. Proposed advances to the dimethacrylate monomers include selectively modifying methacrylate side chains so they are water-compatible, and resistant to hydrolytic [28] and esterase degradation [11,12,13]. Hydroxyethyl methacrylate (HEMA) is the hydrophilic monomer that is most often used in dental adhesives [12]. Incomplete conversion of HEMA can lead to irreversible leaching which undermines adhesive integrity [12]. Advances have included the search for alternative photoinitiators to promote the conversion of hydrophilic monomers [12,29,30,31], new formulations that offer dual polymerization mechanisms [32], and the development of HEMA-free adhesives [33].

A promising strategy for improving dental adhesives involves capitalizing on hydrogen–bond interactions to enhance the polymer network [2,15,19,34]. Monomers containing hydrogen bonding-rich functional groups have been shown to significantly alter intermolecular interactions, leading to enhanced polymer network stability [19,35,36]. The impact of hydrogen bonding capacity on the properties of dental adhesives and composites, as well as approaches for benefiting from hydrogen bonding capacity, are areas of intense interest in adhesive dentistry [2,15,19,34,37,38,39,40].

In our prior investigation, we explored the self-strengthening properties of dental adhesive formulations that are composed of combining silane monomers with methacrylate functionalities [41,42]. Here, we expand this multifunctional monomer system with carbamate functionality to further enhance the properties of dental adhesives.

This study explores the effects of longer chain lengths between silane and methacrylate groups, building upon the strength of our previous work, and incorporates carbamate groups for improving hydrogen bonding interactions. Previous investigators have capitalized on the hydrogen bonding capacity of carbamate groups in methacrylate-based dental composites [43]. To our knowledge, this is the first investigation to capitalize on the hydrogen bonding capacity of carbamates in methacrylate-based dental adhesives. The two-fold objectives of this study are as follows; (i) analyze the impact of longer chain lengths and additional carbamate groups on mobility, crosslink density, stability, and mechanical properties under wet conditions; (ii) compare these properties with methacrylate-based model adhesives containing middle- and short-chained silane monomers.

## 2. Materials and Methods

### 2.1. Materials

**Figure 1:**
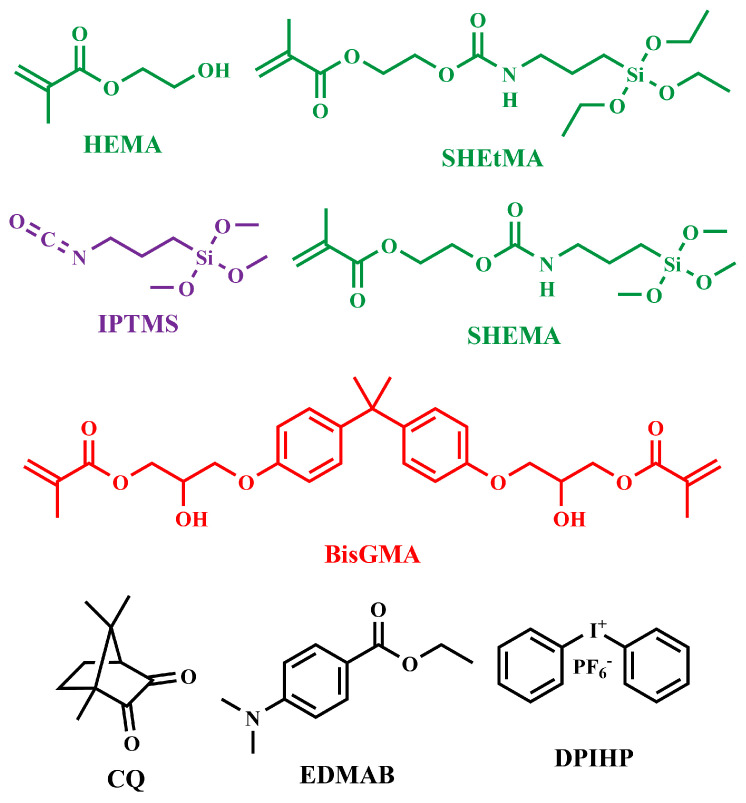
Structures of monomers, molecules, and photoinitiators in formulations.

The monomers 2-hydroxyethyl methacrylate (HEMA) and bisphenol A glycerolate dimethacrylate (BisGMA) were purchased from Sigma-Aldrich Inc. (St. Louis, MO, USA) and used as received without further purification. The photoinitiators diphenyliodonium hexafluorophosphate (DPIHP), camphorquinone (CQ), ethyl-4-(dimethylamino)benzoate (EDMAB), and the catalyst dibutyltin dilaurate (DBTL) were also obtained from Sigma-Aldrich. The silane-containing monomer 4,4-diethoxy-9-oxo-3,10-dioxa-8-aza-4-siladodecan-12-yl methacrylate (SHEtMA) and (3-isocyanatopropyl)trimethoxysilane (IPTMS) were purchased from Gelest Inc. (Morrisville, PA, USA) and Combi-Blocks (San Diego, CA, USA), respectively, and used as received. All other chemicals were reagent-grade and used without further purification. The chemical structures of the monomers, reactants, and photoinitiators are shown in Figure 1.

### 2.2. Synthesis of SHEMA

**Figure 2:**
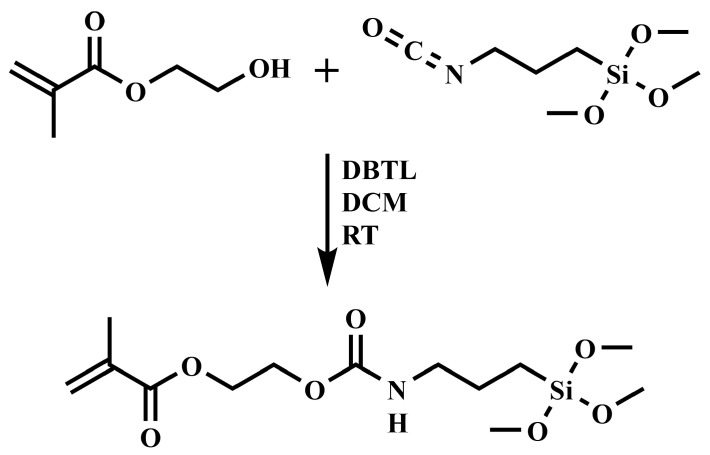
Schematic representation of SHEMA synthesis at room temperature (RT) using dibutyltin dilaurate (DBTL) as catalyst and dichloromethane (DCM) as solvent.

3,3-Dimethoxy-8-oxo-2,9-dioxa-7-aza-3-silaundecan-11-yl methacrylate (SHEMA) was synthesized through a urethane formation reaction catalyzed by dibutyltin dilaurate (DBTL) from 2-hydroxyethyl methacrylate (HEMA) and (3-isocyanatopropyl) trimethoxy silane (IPTMS). The reaction (Figure 2) was carried out by mixing HEMA and IPTMS in a stoichiometric ratio in anhydrous dichloromethane (DCM) at 23 ± 2 °C for 24 h. Specifically, a 250 mL round-bottom flask equipped with a magnetic stirrer and a nitrogen purging system was charged with HEMA (10.0 g, 0.08 mol), DBTL (150 μL), and DCM (100 mL). IPTMS (15.77 g, 0.08 mol), dissolved in 50 mL of DCM, was then added dropwise over the course of 3 h. The progress of the reaction was monitored via FTIR spectroscopy (Frontier FTIR Spectrometer, Perkin-Elmer, Shelton, CT, USA), with particular attention paid to the disappearance of the isocyanate (–NCO) band at 2273 cm^−1^. Upon completion, the solvent DCM was removed under reduced pressure using a rotary evaporator. The final product was obtained as a colorless viscous liquid with an 87% yield. The structure of the resultant product was verified using both FTIR and ^1^H-NMR spectroscopy (AVIIIHD 400 MHz NMR, Bruker, Billerica, MA, USA). The ^1^H-NMR spectrum (CDCl_3_, δ ppm) showed signals at: 0.64 (2H, a), 1.62 (2H, b), 1.95 (3H, c), 3.19 (2H, d), 3.57 (9H, e), 4.31 (4H, f), and 5.59–6.14 (2H, g + h).

### 2.3. Preparation of Adhesive Formulations

A three-component photoinitiator system was used CQ-EDMAB-DPHIP (0.5/0.5/1 wt/wt/wt) for each resin formulation [44,45]. All the mixtures were prepared under amber light in brown glass vials [32]. This procedure was necessary to avoid premature polymerization. For each formulation, HEMA and the organosilanes, SHEtMA (Cb1) or SHEMA (Cb2) were added to amber vials, the photoinitiators were added, and the solutions were mixed thoroughly to obtain homogeneous mixtures. BisGMA was added to the mixture and the formulations were stirred and shaken for 24 h at room temperature (23 ± 2 °C).

The composition of formulations is listed in Table 1. For ease of comparison, the composition and properties of SC4 and SC5 are included in the relevant tables [41]. SC5 contained the short-chain methacryloxymethyltrimethoxysilane (MMeS) while SC4 contained the middle-chain γ-methacryloxypropyltrimethoxysilane (MPS). The formulation composition (30 wt% BisGMA/53 wt% HEMA) was selected based on our group’s previous adhesive studies [41], where this ratio was optimized for bonding to dentin. This composition provided effective dentin infiltration and hybrid layer formation while providing an appropriate matrix to evaluate the performance of novel silane-functionalized monomers. The 30 wt% BisGMA/53 wt% HEMA was deliberately chosen to reflect conditions under which the functional effects of carbamate-based monomers are most likely to manifest, and to enable direct comparison with our previously studied SC4 and SC5 systems.

A comparison of the silane monomers in these formulations from longest chain to shortest chain reveals Cb1 > Cb2 > SC4 > SC5.

### 2.4. Real-Time Double Bond Conversion and Maximum Polymerization Rate

Fourier Transform Infrared Spectroscopy (FTIR) was used to determine the degree of conversion (DC) from monomer to polymer and to assess the polymerization rate [45]. Frontier FTIR Spectrometer (Perkin-Elmer, Shelton, CT, USA) with a spectral resolution of 4 cm^−1^ across a wavenumber range of 650–4000 cm^−1^ was used to record the photopolymerization process in situ and in real-time. The instrument, equipped with Spectrum TimeBase v3.0 software (Perkin-Elmer, Shelton, CT, USA), allowed for continuous spectral acquisition, enabling quantification of DC over time. The transformation of methacrylic C=C double bonds was monitored by analyzing the ratio of absorbance at 1637 cm^−1^ (methacrylic bond) to 1714 cm^−1^ (carbonyl groups). The DC was calculated using the formula DC = (1 − R_P_/R_R_) × 100, where R_P_ represents the post-polymerization band ratio and R_R_ denotes the pre-polymerization band ratio. The average of the final 30 DC measurements, which stabilize at a plateau, was recorded as the DC.

For the experimental preparation, 5–10 μL of the adhesive mixture was applied to the crystal of an attenuated total reflectance (ATR) accessory (Universal ATR Sampling Accessory, Perkin-Elmer, Shelton, CT, USA). To mitigate interference from atmospheric oxygen and moisture, the adhesive was shielded with Mylar film. Following an initial analysis period of 120 s, the sample was irradiated with a commercial visible-light curing unit (Spectrum 800, Dentsply, Charlotte, NC, USA) at peak wavelength of 488 nm and an intensity of 550 mW/cm^2^ for 40 s, during which the infrared spectra were continuously collected over approximately three hours. Each adhesive formula underwent three separate measurements.

The polymerization kinetics were determined by calculating the maximum rate of conversion (R_P_^max^), derived from the first derivative of the DC over time. The values of DC and R_P_^max^ are presented in Table 2 [46].

### 2.5. Water Sorption: Specimen Preparation and Measurement

Round disk samples with dimensions of 1.2 mm in thickness and 4 mm in diameter were prepared for leachate and water sorption studies. Homogeneous adhesive mixtures were carefully loaded into 1 mL cylindrical syringes to avoid air bubble inclusion. The sealed syringes were subjected to visible light photopolymerization for 40 s using an LED light-curing system (LED cure-dome, Prototech, Portland, OR, USA) with a wavelength range of 400–500 nm and an irradiance of 100 mW/cm^2^, followed by storage in the dark for a minimum of 48 h to ensure complete curing [47,48]. The cured syringes were sectioned to the required thickness using an Isomet 1000 Precision Saw (Buehler, Lake Bluff, IL, USA) equipped with a water-cooled diamond disk. These samples underwent a prewash by being submerged in 1 mL of water for seven days at 37 °C, with the prewash water subsequently analyzed for leached species using high-performance liquid chromatography (HPLC) as described in Section 2.7. Post-prewash, the samples were dried under vacuum until a constant mass (*m*_1_) was reached. For the water sorption study, five-disk samples from each formulation were submerged in 2 mL of ultrapure water and weighed at specific intervals: 0, 1, 2, 4, 6, 8, 12, 24, 36, 48, 72, 96, and 120 h, or until they attained a constant mass (*m*_2_). Water sorption was then calculated using Equation (1), where *m*_1_ represents the initial mass of the dried sample and m_2_ the mass at the specified intervals.

(1) $$W_{sp}\%=\frac{m_{2}-m_{1}}{m_{1}}\times 100$$

Water diffusion coefficients were calculated using Fickian diffusion theory for early-stage water sorption (*M_t_/M_∞_* < 0.5) according to Equation (2) below [49]. Where *M_t_* and *M_∞_* are the water mass absorbed at time *t* and at equilibrium, respectively; *D* is the diffusion coefficient; and L is the specimen half-thickness (0.6 mm). Fractional uptake was plotted against t^1/2^, and the diffusion coefficient was extracted from the slope of the linear region using linear regression. This analysis was restricted to the initial sorption phase to ensure purely diffusion-controlled behavior without interference from polymer relaxation or swelling effects that dominate at later stages (*M_t_/M_∞_* > 0.5). Where *M_t_* is the mass of water absorbed at time *t* (mg), *M_∞_* is the mass of water absorbed at equilibrium (mg), *D* is the diffusion coefficient (m^2^/s), *t* is time (s), and *L* is the half-thickness of the disk specimen (m).

(2) $$\frac{M_{t}}{M_{\infty }}=\frac{4}{\sqrt{\pi }}\times {\left(\frac{D\times t}{L^{2}}\right)}^{1/2}$$

### 2.6. Dynamic Mechanical Analysis (DMA)

The viscoelastic behavior of resin formulations Cb1 and Cb2 was evaluated using a dynamic mechanical analyzer (Discovery DMA 850, Waters-TA Instruments, New Castle, DE, USA) equipped with a liquid nitrogen-based cooling system for precise temperature control. Rectangular beam specimens for DMA were prepared with dimensions of 1 mm × 1 mm × 10–15 mm, and four specimens per formulation (Cb1 and Cb2) were analyzed under both dry and wet conditions. Vacuum-dried rectangular beam specimens were analyzed using a standard three-point bending clamp, and the specimens conditioned under water immersion were assessed with a three-point bending submersion clamp. To prepare the rectangular beam specimens, 30–40 μL of resin formulation was injected into borosilicate glass tubes (Vitrocom Technical Glass, 8100 Square VitroTubes™, Mountain Lakes, NJ, USA) at room temperature, followed by photopolymerization in an LED light-curing system for 40 s. For the experimental setup, eight beam specimens per formulation were randomly divided into two groups for comparative analysis under dry and wet conditions. The samples were post-cured in the dark for 48 h, then demolded and incubated in water at 37 °C for seven days to enhance the sol–gel reaction, followed by 48–72 h of drying under vacuum at the same temperature to promote condensation reactions [46]. Beam specimens designated for dry analysis were analyzed after they were exposed to a vacuum environment at 37 °C until a constant mass was achieved. Dynamic mechanical analysis was then conducted over a temperature range of 20–180 °C, with a heating rate of 3 °C/min and a frequency of 1 Hz. Specimens prepared for wet condition testing were first dried under vacuum until reaching equilibrium mass as dry samples, then saturated in ultrapure water at 37 °C until equilibrium mass was again achieved. These water-saturated specimens were subsequently analyzed over a temperature range of 10–90 °C, with a heating rate of 1.5 °C/min and a frequency of 1 Hz with a three-point bend submersion clamp. Throughout all dynamic mechanical tests, a uniform support span of 10 mm was maintained [50].

### 2.7. Leachable Study: Pre-Wash and Pre-Washed Samples Aged in Ethanol

Disk samples (n = 3) of Cb1 and Cb2 were prepared following the protocol outlined in Section 2.5 (Water Sorption). The water from the prewash was collected, and species that leached during the prewash were analyzed using high-performance liquid chromatography (HPLC), as described below. Once the dried disk samples reached a constant mass, they were immersed in 1 mL of ethanol (HPLC Grade). During the initial five days, the storage solution was collected at 24 h intervals and analyzed for leachates. Fresh ethanol was added to the samples following each collection. Sampling continued until the leachate levels reached a plateau, which occurred after seven days for Cb2 and ten days for Cb1. The eluted substances in the ethanol were analyzed quantitatively using HPLC with an LC-2010C HT system (Shimadzu, Lenexa, KS, USA) complemented by Lab Solutions Lite software version 5.132, and a 250 × 4.6 mm column filled with 5 μm C-18 (Luna, Phenomenex Inc., Torrance, CA, USA). The HPLC analysis was performed using a mobile phase of acetonitrile and water containing 0.1% trifluoroacetic acid (TFA), employing a gradient elution from 15/85 (*v*/*v*) to 100/0 (*v*/*v*) over 56 min. Operational parameters included a flow rate of 1 mL/min, UV detection at 208 nm, a 20 μL injection volume, and column temperature maintained at 40 °C. Calibration of the column was executed with standard solutions of BisGMA, HEMA, and EDMAB, generating calibration curves for BisGMA (2.5–250 μg/mL, R^2^ = 0.9995), HEMA (2.5–250 μg/mL, R^2^ = 0.9998), and EDMAB (2.5–100 μg/mL, R^2^ = 0.9999). These calibration curves were used to calculate the concentration of these species in the ethanol solutions based on the chromatographic peak intensities corresponding to the retention times of HEMA (9.3 min), EDMAB (29.0 min), and BisGMA (38.5 min).

### 2.8. Statistical Analysis

The results from the following experiments: water sorption, degree of conversion (FTIR), rate of polymerization, dynamic mechanical analysis (DMA), and accumulative concentration of leachates (HPLC) were analyzed using one-way analysis of variance (ANOVA) together with Tukey’s test at α = 0.05 (Microsoft Excel, Microsoft 365 Suite, Microsoft Corporation, Redmond, WA, USA). Statistical analyses were used to identify significant differences in the means. Throughout the manuscript, significant differences (*p* < 0.05) are indicated by asterisks (*) in tables and explicitly stated in text. All data are presented as mean ± standard deviation.

## 3. Results

**Figure 3:**
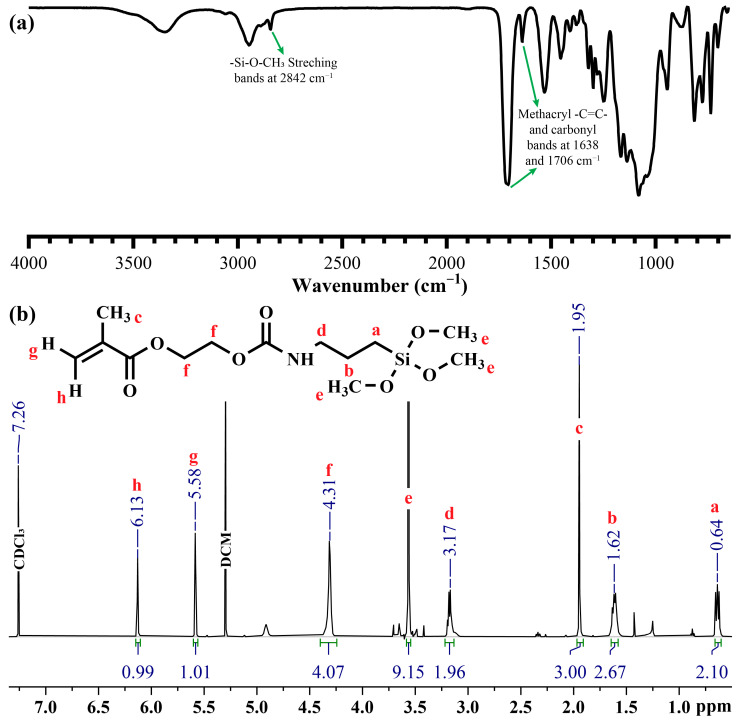
(**a**) FTIR and (**b**) HNMR spectra of SHEMA.

In this study, a new carbamate-functionalized methacrylate monomer, SHEMA, was synthesized by substituting HEMA with methoxy silyl groups via an isocyanate group. The synthesis of SHEMA was achieved through a reaction between the stoichiometric amounts of HEMA and IPTMS. FTIR spectra (Figure 3) confirmed the successful synthesis of SHEMA, with characteristic peaks indicating the substitution process. The appearance of a new peak at 2842 cm^−1^ corresponds to the methoxy silane group, and the disappearance of the isocyanate (–NCO) band at 2273 cm^−1^ supports the structure. This substitution is indicative of the formation of the carbamate linkage in the SHEMA monomer. The ^1^H-NMR spectrum (CDCl_3_, δ ppm) provided further confirmation of the SHEMA structure, showing signals at: 0.64 (2H, a), 1.62 (2H, b), 1.95 (3H, c), 3.17 (2H, d), 3.57 (9H, e), 4.31 (4H, f), and 5.58–6.13 (2H, g+h). These NMR data are consistent with the expected chemical shifts for the synthesized monomer, supporting the successful incorporation of the methoxy silane group via carbamate linkage.

**Figure 4:**
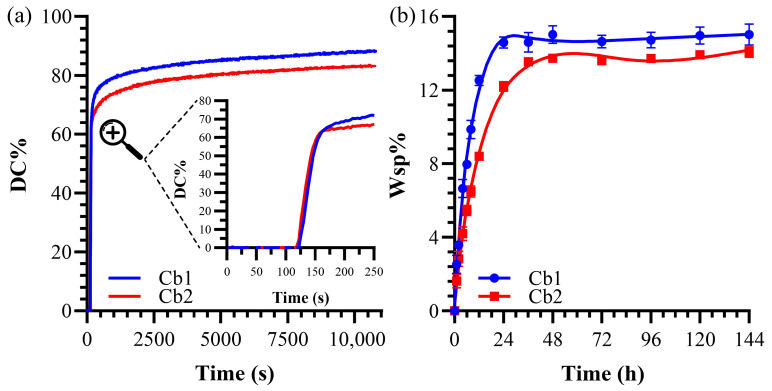
(**a**) Degree of conversion of formulations. The magnified inset (indicated by dashed lines and magnifier symbol) shows the detail of the first 250 seconds. (**b**) Water sorption values of formulations.

The compositions of the formulations are listed in Table 1. The degree of conversion (DC) and maximum polymerization rate (R_P_^max^/[M]) for resin formulations are provided in Table 2. Formulations consist of the same amount of BisGMA (30 wt%) as crosslinker, photoinitiator system (2 wt%—CQ-EDMAD-DPIHP:0.5-0.5-1), and HEMA (53 wt%). The DC% of Cb1 (88.20 ± 2.3%) is statistically significantly greater (*p* < 0.05) than Cb2 (83.24 ± 1.8%). The degree of conversion (DC) as a function of time is depicted in Figure 4a. Cb1 contains SHEtMA as long-chain silane monomer in comparison to SHEMA. The maximum polymerization rate (R_P_^max^ (1/[M])) of Cb1 is significantly less than that of Cb2, with values of 2.42 ± 0.5 and 2.88 ± 0.3, respectively.

The solid copolymer resin disks showed a gradual increase in water sorption, stabilizing between 36 and 48 h of storage at 37 °C (Figure 4b). Significant differences in water sorption were noted between Cb1 and Cb2, with measurements of 15.02 ± 0.6% and 14.07 ± 0.3%, respectively (Table 2). It is known that formulations with a reduced content of the hydrophobic BisGMA crosslinker tend to absorb more water. However, the observed difference between Cb1 and Cb2, despite having the same proportion of the crosslinker, indicates possible reactivity differences between SHEtMA and SHEMA. The reduced water sorption in Cb2 suggests a relative increase in hydrophobicity, likely due to a more extensive crosslinked network and a higher number of Siloxane crosslinks formed through the sol–gel reaction.

For ease of comparison, the values of the formulations with short-chain and medium-chain silane monomers from our previous investigation [41] are provided in Table 3.

The mechanical properties of the solid copolymer resin rectangular beam samples were assessed under both dry and wet conditions. Distinct differences were observed between Cb1 and Cb2. Values have been shown in Table 4 for dry analysis and Table 5 water-submersed analysis.

**Figure 5:**
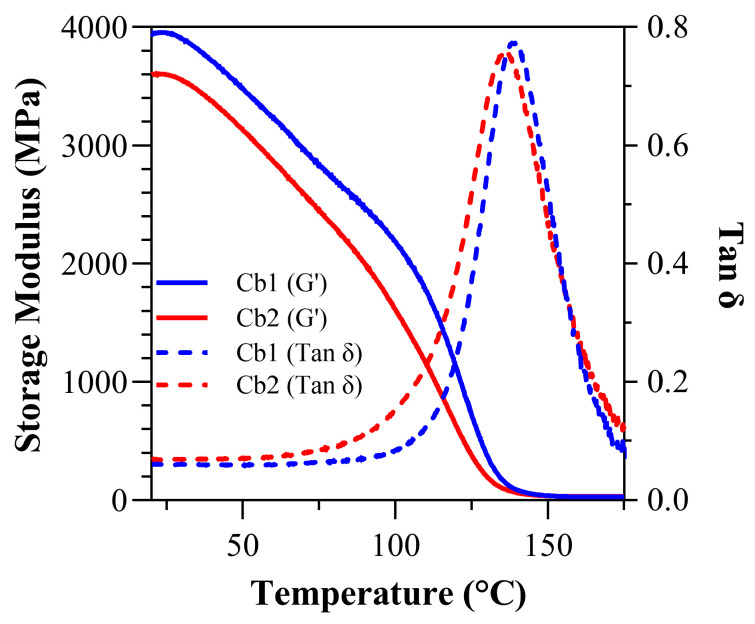
Under dry conditions: tan δ vs. temperature curves and storage modulus vs. temperature curves for Cb1 and Cb2.

Under dry conditions at 37 °C, the storage modulus (GPa) for Cb1, 3.78 ± 0.11, is significantly greater (*p* < 0.05) than Cb2, 3.43 ± 0.18 (Figure 5). At 70 °C, the storage modulus (GPa) decreased to 2.97 ± 0.11 and 2.59 ± 0.20 for Cb1 and Cb2, respectively. The dry rubbery modulus (GPa) was 0.029 ± 0.00 (28.75 ± 0.71 MPa) and 0.032 ± 0.00 (31.60 ± 2.67 MPa) for Cb1 and Cb2, respectively. There is no statistically significant difference between the dry rubbery modulus values. The dry glass transition temperature (T_g_) for Cb1 was significantly greater than Cb2 at 138.99 ± 0.60 °C and 135.84 ± 1.78 °C, respectively. The dry calculated inverse ratio (ζ) was 5.74 × 10^−6^ (Pa·K^−1^) for Cb1, which is slightly higher than 5.07 × 10^−6^ (Pa·K^−1^) for Cb2. The dry full-width-at-half-maximum (FWHM) was 30.70 ± 0.70 for Cb1 and 34.03 ± 3.37 for Cb2, while the dry loss factor was 0.7736 ± 0.01 for Cb1 and 0.7692 ± 0.02 for Cb2 (Table 4). There are no statistical differences (*p* > 0.05) in FWHM and calculated inverse ratios.

**Figure 6:**
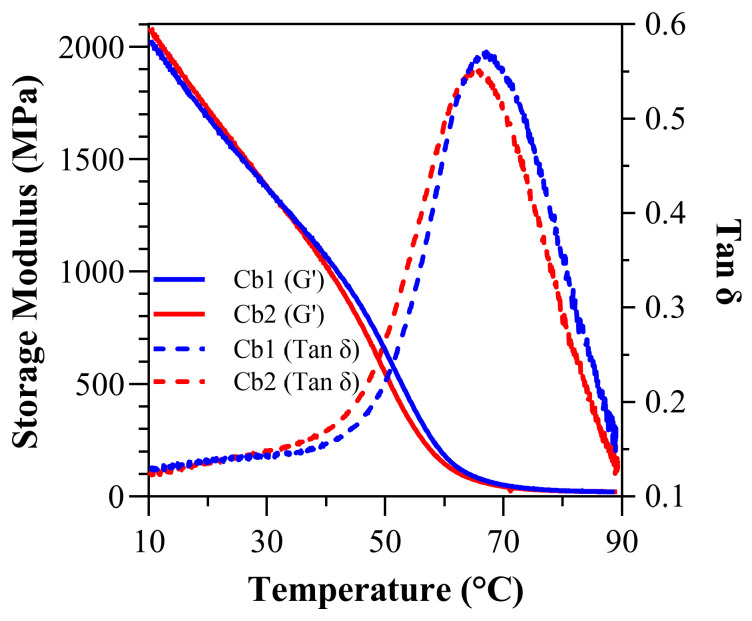
Under submersed conditions: tan δ vs. temperature curves and storage modulus vs. temperature curves for Cb1 and Cb2.

Under water-submersed conditions (Figure 6 and Table 5), at 37 °C, the storage modulus (GPa) for Cb1 was significantly greater (*p* < 0.05) than Cb2 at 1.16 ± 0.00 (1163.20 ± 6.50 MPa) and 1.03 ± 0.00 (1027.60 ± 40.8 MPa), respectively. At 70 °C, the wet storage modulus (GPa) dropped precipitously, and the values were comparable 0.05 ± 0.00 (51.28 ± 4.20 MPa) and 0.04 ± 0.00 (37.02 ± 8.90 MPa) GPa for Cb1 and Cb2, respectively. The wet rubbery modulus for both Cb1 and Cb2 was 0.02 ± 0.00 GPa (20.69 ± 0.40 MPa and 19.16 ± 1.10 MPa, respectively). The wet T_g_ was measured at 67.93 ± 0.7 °C for Cb1 and 65.55 ± 0.9 °C for Cb2 (*p* < 0.05). The calculated inverse ratio (ζ) under submersed conditions was 4.16 × 10^−6^ (Pa^−1^·K) for Cb1 and 4.50 × 10^−6^ (Pa^−1^·K) for Cb2. The wet FWHM was 28.98 ± 0.79 for Cb1 and 30.32 ± 0.3 for Cb2, while the wet loss factor was 0.5760 ± 0.02 for Cb1 and 0.5570 ± 0.00 for Cb2 (Table 5).

In water-submersed DMA, there is a reduction in storage modulus compared to dry conditions. For Cb1 and Cb2, this reduction is 69.24% and 70.06% at 37 °C, respectively. In the formulations from our previous study, also with 30 wt% BisGMA, the reductions are 73.06% and 65.26%, respectively, for SC4 and SC5 [41]. A comparison of these formulations reveals that with almost identical composition except for alkoxysilane-containing methacrylate monomer, the chain length from longest to shortest Cb1 > Cb2 > SC4 > SC5. There is a known advantage in the shortest chain silane monomer with respect to the mechanical property. Although the longer-chained monomers typically result in reduced polymer stiffness, in this study, the additional hydrogen-bonding in SHEMA and SHEtMA from Cb2 and Cb1, respectively, led to formulations that exhibit greater resilience as compared to middle-chain MPS-containing SC4. Also, since the tan δ peaks were reached under submersed conditions for Cb1 and Cb2, loss factor change can also be calculated for these formulations, which are −25.54% and −27.59%, respectively. These changes suggest reduced flexibility in water-submersed conditions; these results potentially reflect the impact of increased hydrogen bonds (Table 6).

The wet-state modulus values must be interpreted within the context of the testing conditions. At physiologically relevant temperature (37 °C), both Cb1 and Cb2 maintained storage moduli exceeding 1 GPa under full water submersion, well above clinical performance thresholds. The lower modulus values at 70 °C represent the rubbery plateau region, accessible in this study due to the exceptional network stability provided by carbamate functionalities. Importantly, the continuous water submersion at elevated temperature represents aggressive conditions that generally exceed cyclic exposure experienced in vivo. These results suggest that these formulations will provide enhanced performance under clinical conditions. The demonstration of measurable rubbery modulus under submersed conditions, previously unachievable with our SC4/SC5 systems, represents a significant advancement in network stability for this class of materials.

**Figure 7:**
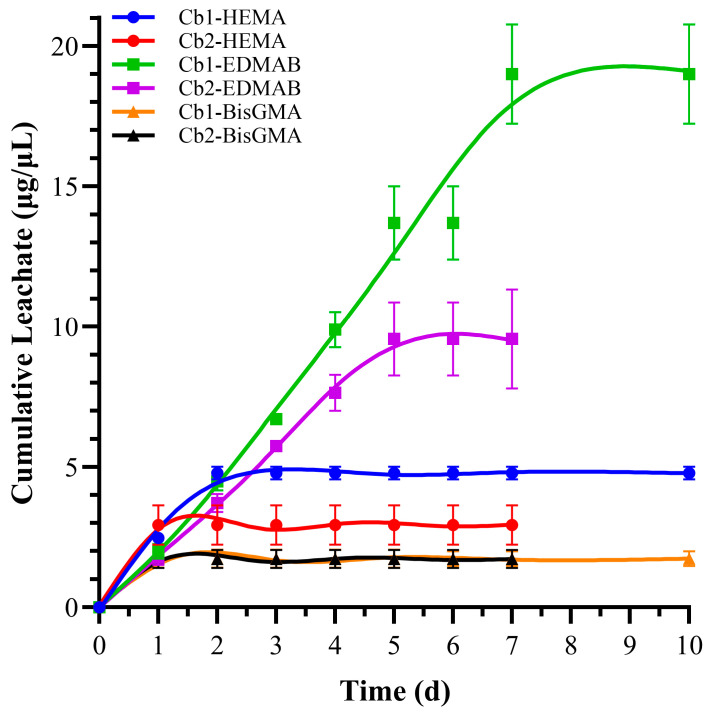
Time-dependent cumulative leachate concentrations of HEMA, BisGMA, and EDMAB from Cb1 and Cb2 formulations during ethanol incubation. Data points represent total accumulated concentration at each time interval. Note that Cb2 formulation reaches equilibrium plateau by day 7, while Cb1 continues releasing until day 10, suggesting differences in network tortuosity and diffusional resistance.

The leachate analysis of the copolymer resin disk samples revealed significant differences between Cb1 and Cb2 after ethanol incubation, as determined by high-performance liquid chromatography (HPLC) [45]. Values have been indicated in Table 7 and presented in Figure 7.

All disks for both the leachate and water sorption study were incubated in ultrapure water for seven days [41]. The prewash water was analyzed using HPLC. The prewash HEMA leachate for Cb1 was 36.93 ± 6.7 µg/mL, while for Cb2 it was 32.95 ± 0.7 µg/mL, with no statistically significant difference between these values. Cumulative HEMA leachate levels after ethanol incubation were 4.78 ± 0.2 µg/mL for Cb1, which is approximately 1.5 times higher than the 2.93 ± 0.7 µg/mL observed for Cb2, indicating statistically significant differences between the two formulations (*p* < 0.05). For cumulative BisGMA leachate, there was no significant statistical difference for Cb1 and Cb2 at 1.73 ± 0.3 µg/mL and 1.72 ± 0.3 µg/mL, respectively. The cumulative EDMAB leachate after ethanol incubation was significantly higher in Cb1, at 19.01 ± 1.8 µg/mL, compared to 9.56 ± 0.6 µg/mL for Cb2. This represents approximately a twofold increase in leachate levels for Cb1 relative to Cb2, with statistically significant differences in the formulations (*p* < 0.05). Additionally, HPLC analysis detected some peaks corresponding to oligomers; however, no leachate signals were detected for either SHEMA or SHEtMA.

These findings highlight notable differences in the leachate profiles of Cb1 and Cb2 after ethanol incubation, suggesting that Cb1 leached more HEMA and EDMA under the conditions in this study. The absence of SHEMA and SHEtMA leachate signals indicates complete incorporation within the polymer matrix.

## 4. Discussion

### 4.1. Polymerization Behavior

The degree of conversion (DC%) and polymerization rate (R_P_^max^) are critical indicators of the performance of adhesive formulations [32]. In this study, Cb1 (SHEtMA) exhibited a DC of 88.20 ± 2.27 and an R_P_^max^ of 2.42 ± 0.5, while Cb2 (SHEMA) showed a DC of 83.24 ± 1.84 and an R_P_^max^ of 2.88 ± 0.3. Despite the molecular similarity and comparable chain lengths, the DC values differ significantly (*p* < 0.05), with Cb1 showing higher DC (Figure 4a).

This disparity can be attributed to the greater potential for sol–gel reactions of methoxy silanes compared to ethoxy silanes [51,52]. The faster polymerization rate observed in Cb2 could cause the auto-acceleration (Trommsdorff–Norrish effect), a phenomenon in free-radical polymerization systems, where localized radicals increase in viscosity, slow termination reactions, leading to a rapid increase in the overall rate of reaction [53,54]. This effect can cause a reaction runaway and alter the characteristics of the polymers produced, possibly explaining the lower DC for Cb2. Additionally, the lower DC for Cb2 may be due to the easier formation of Si-O-Si bonds through methoxy silanes compared to ethoxy silanes.

In comparison, formulations SC4 and SC5 from our former study [41] exhibited DC values of 79.7 ± 0.20% and 75.6 ± 0.15%, respectively (Table 3). SC4 consists of MPS, a middle-chain monomer, while SC5 has MMeS, the smallest silane monomer that was used in our studies. These results indicate that as the monomers get smaller, the polymerization rate increases but the degree of conversion decreases due to the Trommsdorff–Norrish effect. Higher DC values in Cb1 and Cb2 compared to SC4 and SC5 underscore the impact of monomer size on the balance between polymerization rate and degree of conversion in adhesive formulations.

### 4.2. Water Sorption

The investigation into water sorption properties across different polymer formulations provides critical insights into how crosslinker weight percentages and specific functional groups impact material performance [50,55]. Formulations incorporating 30 wt% BisGMA show consistent water sorption percentages, ranging from 13–14% to 15–16%, irrespective of variations in other monomer components [41]. A focused analysis of Cb1 and Cb2 formulations highlights the pivotal role of methoxy silane groups in determining water sorption characteristics. Methoxy silane groups in the SHEMA component of Cb2 enhance the sol–gel reaction efficiency, leading to a more extensive formation of Si-O-Si bonds [51]. This structural attribute significantly reduces the hydrophilicity of Cb2 compared to Cb1, resulting in a statistically significantly lower water sorption percentage (*p* < 0.05). Specifically, Cb2 exhibits a water sorption value of 14.07 ± 0.29%, whereas Cb1 shows a higher value of 15.02 ± 0.57% (Figure 4b). The results from our former studies showed that if hydrophobicity is dominant, even an increased number of Si-O-Si bonds did not significantly reduce water sorption capacity [45]. However, as the basic networks’ hydrophilicity increases, the number of silane bonds in the resin network may have more pronounced effects on the hydrophilicity of the polymer material. The difference between W_sp_% values of Cb1 and Cb2 is an important indicator of this observation (Table 2).

The Si-O-Si network formed during the sol–gel process is essential in enhancing the material’s hydrophobic properties. This robust three-dimensional network decreases the material’s affinity for water, which is evident when comparing Cb1 and Cb2. The methoxy silane groups in Cb2 facilitate a denser and more extensive Si-O-Si network, which may be due to the higher reactivity of methoxy silane groups as compared to ethoxy silanes [52]. This extensive network not only reduces water uptake but also enhances the mechanical stability of the polymer which suggests that Cb2 may be more suitable for applications where moisture resistance is crucial.

In comparison with previous 30% BisGMA formulations, such as SC4 and SC5, the longer chain length and hydrogen bonding capacity in formulations like Cb1 contribute to higher water sorption. Despite the minor differences between SC4 and SC5, Cb1 exhibits higher water sorption, suggesting that while chain length and hydrogen bonding influence water sorption, the superior sol–gel reaction of Cb2, driven by methoxy silane groups, results in lower water sorption. This indicates that the sol–gel process in Cb2 effectively overcomes the potential hydrophilicity introduced by hydrogen bonding. The statistically significant difference in water sorption between Cb1 and Cb2, despite their similar components, underscores the critical role of the sol–gel reaction facilitated by methoxy silane groups.

Formulations SC4 and SC5, which show water sorption percentages comparable to Cb2, likely benefit from the intensive formation of Si-O-Si bonds, supporting the hypothesis that the methoxy silane groups in Cb2 significantly enhance its sol–gel reaction and water sorption properties. This reinforces the notion that specific functional groups within the polymer network critically influence water sorption characteristics. The comparable performance of SC4 and SC5 with Cb2 suggests that the presence of similar functional groups can achieve comparable water resistance, emphasizing the versatility and importance of sol–gel chemistry in designing relatively hydrophobic polymers.

By integrating quantitative diffusion modeling and indirect validation through structural and mechanical evidence, the Fickian diffusion analysis revealed a notably lower water diffusion coefficient for Cb2 (5.93 × 10^−13^ m^2^/s) compared to Cb1 (1.07 × 10^−12^ m^2^/s). The combination of significantly lower water diffusion coefficient (45% reduction) decreased equilibrium water sorption, and enhanced mechanical stability under wet conditions. The results collectively demonstrate that Cb2 exhibits superior hydrophobic characteristics compared to Cb1. These differences are attributed to the more efficient sol–gel reaction of methoxy silane groups, leading to a denser Si-O-Si network that restricts water penetration and plasticization.

In summary, the incorporation of methoxy silane groups in Cb2 leads to a superior sol–gel reaction, significantly reducing water sorption by forming an extensive Si-O-Si network. This structural feature enhances the hydrophobic properties of the material, making Cb2 a more effective formulation for applications requiring low water sorption. These findings underscore the essential role of functional groups in influencing the sol–gel reaction and the water sorption properties of polymer formulations. The study highlights that optimizing functional group chemistry and crosslinker composition can lead to significant improvements in material performance, particularly in environments where moisture resistance is critical.

### 4.3. Leachate Properties

After prewashing the disk samples in water for seven days, the prewash water was analyzed using HPLC. The results indicated that there were no detectable levels of BisGMA, EDMAB, or SHEMA/SHEtMA leachates in the prewash water samples. The absence of BisGMA, EDMAB, and SHEMA/SHEtMA leachates suggests a high degree of polymerization and cross-linking for these components, resulting in their strong incorporation into the polymer matrix. However, due to the hydrophilic nature of HEMA, a significant amount of HEMA leachates was found in the prewash water. The significant amount of HEMA leachate indicates residual unreacted monomer in the polymer samples.

The time-dependent leachate profiles (Figure 7) reveal distinct release kinetics for different components. The cumulative presentation allows identification of plateau behavior, which occurs earlier for Cb2 (7 days) than Cb1 (10 days), suggesting that the denser network in Cb2 not only releases less total material but also reaches equilibrium more rapidly. The non-linear cumulative curves indicate that release is initially diffusion-controlled but progressively slows as readily accessible material is depleted. While formal kinetic modeling was not performed in this comparative study, future work will employ release models to extract diffusion parameters and predict long-term degradation behavior.

Although Cb1 had a higher HEMA leachate concentration (36.93 ± 6.7 μg/mL) compared to Cb2 (32.95 ± 0.7 μg/mL), the difference between the two formulations was not statistically significant (*p* > 0.05). These results suggest that the difference between methoxy and ethoxy silane groups does not significantly affect the amount of unreacted HEMA that is leached during the prewash process. This indicates that the functional group differences between SHEtMA (ethoxy silane) and SHEMA (methoxy silane) do not play a major role in the initial leaching behavior of HEMA. Since the monomer weight ratios are the same for the two formulations, there is no pronounced difference in the weight percentages of the leachates as well.

Ethanol solutions were collected and refreshed daily until the leachate levels plateaued. For Cb2, the EDMAB leachate concentration reached a plateau after seven days, measuring 9.56 ± 0.61 μg/mL, which is statistically significantly lower than the concentration observed for Cb1, which plateaued at 19.01 ± 1.77 μg/mL after ten days. The differences in the EDMAB leachate values suggest that the denser network of Cb2 led to reduced leaching of this component. BisGMA leachate values were comparable for the two formulations. Cb1 exhibited a twofold increase in the leaching of both HEMA and EDMAB compared to Cb2 (*p* < 0.05). This significant increase in leachate levels suggests a less effective polymer network with higher amounts of unreacted monomers in Cb1.

Notably, no leachates of SHEMA or SHEtMA were detected in the study, which suggests effective integration of carbamate monomers within the resin network through FRP and sol–gel reaction. The lack of SHEMA or SHEtMA leachates may reflect the effect of extra hydrogen bonding donor and acceptor units in the carbamate functionalities of the SHEMA and SHEtMA monomers. The effective integration of these monomers suggests that the sol–gel reaction as well as the carbamate functionalities contribute to a stable and well-formed polymer network, reducing the leachate potential of these specific monomers (Figure 7).

The leachate analysis revealed that both Cb1 and Cb2 had lower EDMAB leachate levels compared to SC4 and SC5, suggesting better integration and stability of the network, thus supporting the hypothesis that longer chain monomers and additional hydrogen bonding will increase structural integrity of resins. The difference in chain lengths between SC4, SC5, and the Cb formulations indicates that longer chains (in Cb1 and Cb2) might improve network interactions and reduce heterogeneity, thereby enhancing the overall stability and reducing leachates.

The differences in the degree of conversion (DC) should also be considered when comparing leachate values of SC4, SC5, and the Cb formulations. SC4 has a middle-chain monomer, while SC5 has a short-chain monomer [41]. Although network interactions in formulations with longer chain monomers such as Cb1 and Cb2 may lead to reduce heterogeneity (based on the FWHM values), the primary reason for reduced leachate compared to SC4 and SC5 may be related to the increased DC. The increased DC of Cb1 and Cb2 suggests a decreased number of unreacted monomers trapped in the network and thus, reduced leachates in ethanol. Under the conditions of this investigation, the increased chain length of the Cb formulations as compared to middle- (SC4) and short-chain (SC5) silane monomers led to reduced leachates. Overall, the study indicates that the formulation and specific chemical functionalities within the polymer network significantly influence the leachate properties, with longer chain monomers and effective network integration leading to reduced leachate levels and enhanced material stability.

### 4.4. Mechanical Properties

Dynamic Mechanical Analysis (DMA) of the Cb1 and Cb2 formulations, which consist of 30 wt% BisGMA and 53 wt% HEMA, reveals significant insights into their mechanical properties. Both formulations incorporate different silane functional groups, with Cb1 containing SHEtMA (ethoxy silane) and Cb2 containing SHEMA (methoxy silane). The structural difference between these groups is known to influence their efficiency in sol–gel reactions, potentially affecting the mechanical and structural properties of the formulations [50,51,55].

At 37 and 70 °C, Cb1 demonstrates higher storage modulus values than Cb2, indicating increased stiffness at these temperatures under dry conditions. Specifically, Cb1’s storage modulus at 37 °C is 3.78 ± 0.11 GPa and at 70 °C is 2.97 ± 0.11 GPa, compared to Cb2’s 3.43 ± 0.18 GPa at 37 °C and 2.59 ± 0.20 GPa at 70 °C. Regarding the rubbery modulus, Cb2 is slightly higher than Cb1 (0.031 ± 0.00 GPa (31.60 ± 2.60 MPa) vs. 0.028 ± 0.00 GPa (28.75 ± 0.71 MPa), respectively), although this difference is not statistically significant (*p* > 0.05). The glass transition temperature (T_g_) is also higher for Cb1 (138.99 ± 0.60 °C) compared to Cb2 (135.84 ± 1.78 °C), indicating that Cb1 maintains its structural integrity at higher temperatures under dry conditions.

The calculated crosslink ratio suggests that Cb2 has a more crosslinked network, as evidenced by its lower crosslink ratio as compared to Cb1. The higher FWHM value for Cb2 (34.03 ± 3.37) compared to Cb1 (30.70 ± 0.70) suggests greater heterogeneity.

Under water-submersed conditions, both Cb1 and Cb2 exhibit a significant decrease in storage modulus, but the decrease is less than SC4 (a middle-chained silane monomer) [41]. The reduction in storage modulus for Cb1 and Cb2 is 69.24% and 70.06%, respectively, whereas SC4 showed a loss of 73.06%. Under water-submersed conditions, the short-chained silane monomer (SC5) showed a 65.26% decrease in storage modulus. The results with SC5 may reflect the pronounced benefits of short-chained silane monomers.

The Cb formulations showed less loss in storage modulus under water-submersed conditions than the middle-chained silane monomer formulation SC4. Also, for Cb1 and Cb2, the decrease in loss factor values in water-submersed conditions is 25.54% and 27.59% which also indicates the highly effective impact of hydrogen bonding through carbamate functionality in moist conditions (Table 6). Under conditions where moisture is more pronounced, hydrogen bonding capacity will increase, and this will reflect on the reduced flexibility of the network, which can be inferred from the loss factor changes. This indicates that the hydrogen bonding in Cb1 and Cb2 networks is more influential than the decrease in the mechanical properties under water-submerged conditions attributable to chain lengths. Thus, hydrogen bonding contributes to enhanced mechanical stability in Cb1 and Cb2.

Overall, the DMA highlights that while both Cb1 and Cb2 formulations exhibit robust mechanical properties, under dry conditions Cb1 tends to perform better in terms of storage modulus and glass transition temperature. These results suggest that under dry conditions, Cb1 may be suitable for applications that require higher mechanical strength and stability. The influence of hydrogen bonding is particularly significant under wet conditions, contributing to the reduced loss in mechanical properties and emphasizing the importance of carbamate functionality in these formulations.

## 5. Conclusions

The formulations containing the carbamate-functionalized long-chained silane monomers showed superior retention of storage moduli in wet conditions compared to formulations with middle-chained silane monomers. This improvement is attributed to the hydrogen bonds in Cb1 and Cb2 which reinforced the polymer network, enhanced the mechanical properties, and restricted chain flexibility.

Further investigations are required to refine and optimize these dental adhesive formulations. Additional studies are required to determine the biocompatibility of formulations containing these novel, multi-functional monomer systems. The long-term performance and adhesive/dentin interfacial properties must be determined in future investigations.

The methacrylate-based model dental adhesives containing carbamate-functionalized long-chain silane monomers exhibited significantly improved mechanical properties and hydrolytic resistance. The results offer promising advancements in the development of durable dental adhesives. The results contribute to our library of strategic approaches for extending the lifespan of dental composite restorations.

## Figures and Tables

**Table 1:** Chemical composition of the formulations.

Ratio%	Cb1	Cb2	SC4 [41]	SC5 [41]
HEMA	53	53	53	53
BisGMA	30	30	30	30
SHEMA ^1^	-	15	-	-
SHEtMA ^1^	15	-	-	-
MPS ^1^	-	-	15	-
MMeS ^1^	-	-	-	15
3-PI ^2^	2	2	2	2
Total	100	100	100	100

^1^ MW_SHEMA_: 335.43 g/mol, MW_SHEtMA_: 377.51 g/mol, MW_MPS_: 248.35 g/mol, MW_MMeS_: 220.30 g/mol. ^2^ Photoinitiators-CQ-EDMAD-DPIHP: 0.5-0.5-1.

**Table 2:** Values of degree of conversion (DC%), maximum polymerization rate (R_P_^max^/[M]), water sorption (W_sp_%), and diffusion coefficient D (m^2^/s).

Formulation	DC%	R_P_^max^ (1/[M])	W_sp_%	D (m^2^/s)
Cb1	88.20 ± 2.3	2.42 ± 0.5	15.02 ± 0.6	1.07 × 10^−12^
Cb2	83.24 ± 1.8 *	2.88 ± 0.3 *	14.07 ± 0.3 *	5.93 × 10^−13^

* indicates a significant difference at *p* < 0.05.

**Table 3:** Properties of SC formulations from Demirel et al. [41].

Values	SC4	SC5
DC%	79.7 ± 0.2	75.6 ± 0.2
R_P_^max^	2.1 ± 0.2	2.8 ± 0.1
W_sp_%	14.23 ± 0.1	14.38 ± 0.5
FWHM	48.62 ± 1.1	66.06 ± 2.1
Dry Storage Modulus (GPa)	4.21 ± 0.24 (37 °C) 3.47 ± 0.19 (70 °C)	4.40 ± 0.18 (37 °C) 3.62 ± 0.15 (70 °C)
Wet Storage Modulus (GPa)	1.14 ± 0.08 (37 °C) 0.32 ± 0.03 (70 °C)	1.49 ± 0.05 (37 °C) 0.57 ± 0.0.2 (70 °C)
T_g_ (°C)	159.58 ± 0.8	170.31 ± 0.9
Calculated Inverse Ratio, ζ	1.97 ± 0.1 × 10^−6^ Pa^−1^ K	0.52 ± 0.0 × 10^−6^ Pa^−1^ K
Cumulative HEMA Leachate (μg/mL)	40.57 ± 1.5	56.49 ± 2.2
Cumulative BisGMA Leachate (μg/mL)	5.01 ± 0.6	1.44 ± 0.2
Cumulative EDMAB Leachate (μg/mL)	31.86 ± 1.6	22.22 ± 1.0
Cumulative HEMA Leachate (wt%)	0.31 ± 0.0	0.43 ± 0.0
Cumulative EDMAB Leachate (wt%)	25.49 ± 1.3	17.78 ± 0.8
Cumulative BisGMA Leachate (wt%)	0.07 ± 0.0	0.02 ± 0.0

**Table 4:** Values of the mechanical properties of vacuum dried samples at various temperatures.

Values	Cb1	Cb2
Dry Storage Modulus (GPa)	3.78 ± 0.11 (37 °C) 2.97 ± 0.11 (70 °C)	3.43 ± 0.18 (37 °C) * 2.59 ± 0.20 (70 °C) *
Dry Rubbery Modulus (GPa)	0.029 ± 0.00	0.032 ± 0.00
Dry T_g_ (°C)	138.99 ± 0.60	135.84 ± 1.78 *
Dry Calculated Inverse Ratio, ζ	5.74 × 10^−6^ (Pa^−1^ K)	5.07 × 10^−6^ (Pa^−1^ K) *
Dry FWHM	30.70 ± 0.70	34.03 ± 3.37
Dry Loss Factor	0.7736 ± 0.01	0.7692 ± 0.02

* indicates a significant difference at *p* < 0.05.

**Table 5:** Values of the mechanical properties of water-submersed samples at various temperatures.

Values	Cb1	Cb2
Wet Storage Modulus (GPa)	1.16 ± 0.00 (37 °C) 0.05 ± 0.00 (70 °C)	1.03 ± 0.00 (37 °C) * 0.04 ± 0.00 (70 °C)
Wet Rubbery Modulus (GPa)	0.021 ± 0.00	0.019 ± 0.00
Wet T_g_ (°C)	67.93 ± 0.7	65.55 ± 0.9
Wet Calculated Inverse Ratio (ζ)	4.16 × 10^−6^ (Pa^−1^ K)	4.50 × 10^−6^ (Pa^−1^ K)
Wet FWHM	28.98 ± 0.79	30.32 ± 0.3
Wet Loss Factor	0.5760 ± 0.02	0.5570 ± 0.00

* indicates a significant difference at *p* < 0.05.

**Table 6:** Change ratios of formulations between dry and water-submersed conditions.

Formulations	Loss in Storage Modulus at 37 °C	Change in Loss Factor (Tan δ)
Cb1	69.24%	−25.54%
Cb2	70.06%	−27.59%
SC4 [41]	73.06%	N/A
SC5 [41]	65.26%	N/A

**Table 7:** Values of prewash and cumulative ethanol degradation leachates for Cb1 and Cb2.

Values	Cb1	Cb2
Prewash HEMA Leachate (μg/mL)	36.93 ± 6.7	32.95 ± 0.7
Cumulative HEMA Leachate (μg/mL)	4.78 ± 0.2	2.93 ± 0.7 *
Cumulative BisGMA Leachate (μg/mL)	1.73 ± 0.3	1.72 ± 0.3
Cumulative EDMAB Leachate (μg/mL)	19.01 ± 1.8	9.56 ± 0.6 *
Prewash HEMA Leachate (wt%)	0.28 ± 0.1	0.25 ± 0.0
Cumulative HEMA Leachate (wt%)	0.04 ± 0.0	0.02 ± 0.0 *
Cumulative BisGMA Leachate (wt%)	0.02 ± 0.0	0.02 ± 0.0
Cumulative EDMAB Leachate (wt%)	15.20 ± 1.44	7.65 ± 0.5 *

* indicates a significant difference at *p* < 0.05.

## Data Availability

The original contributions presented in this study are included in the article. Further inquiries can be directed to the corresponding authors.
